# Nutritional adequacy of diets containing growing up milks or unfortified cow's milk in Irish children (aged 12–24 months)

**DOI:** 10.3402/fnr.v57i0.21836

**Published:** 2013-12-02

**Authors:** Janette Walton, Albert Flynn

**Affiliations:** School of Food & Nutritional Sciences, University College Cork, Cork, Ireland

**Keywords:** growing up milks, toddler milks, cow's milk, iron, vitamin D, children

## Abstract

**Background:**

Growing up milks (GUM) are milk-based drinks with added vitamins and minerals intended for children aged 12–36 months. Few data are available on the consumption of GUM and their role in the diets of young children.

**Objective:**

To determine the nutritional adequacy of two groups of 12–24-month-old Irish children by type of milk consumption (consumers or non-consumers of GUM).

**Design:**

Using data from a cross-sectional study of Irish children, the National Pre-School Nutrition Survey (2010–2011), two groups of children were defined. The groups included children aged 12–24 months with an average daily total milk intake of at least 300 g and consuming GUM (≥100 g/day) together with cow's milk (*n*=29) or cow's milk only (*n*=56).

**Results:**

While average total daily energy intakes were similar in both consumers and non-consumers of GUM, intakes of protein, saturated fat, and vitamin B12 were lower and intakes of carbohydrate, dietary fibre, iron, zinc, vitamins C and D were higher in consumers of GUM. These differences in nutrient intakes are largely attributable to the differences in composition between GUM and cow's milk. For both consumers and non-consumers of GUM, intakes of carbohydrate and fat were generally in line with recommendations while intakes of protein, dietary fibre and most micronutrients were adequate. For children consuming cow's milk only, high proportions had inadequate intakes of iron and vitamin D; however, these proportions were much lower in consumers of GUM.

**Conclusions:**

Consumption of GUM reduced the risk of inadequacies of iron and vitamin D, two nutrients frequently lacking in the diets of young children consuming unfortified cow's milk only.

Growing up milks (GUM) are milk-based drinks with added vitamins and minerals intended for children aged 12–36 months. The regulatory status of GUM is currently under review in the European Union (EU) in the context of the proposed revision of Directive 2009/39/EC of the European Parliament and of the Council of 6 May, 2009, on Foodstuffs intended for Particular Nutritional Uses (PARNUTS). The European Commission has recently requested the European Food Safety Authority (EFSA) to provide advice on the need for such milks for young children and their nutritional composition (1).

There are few data available on the role of GUM in the diets of young children in Europe. A recent study in French children (1–2 years) (2) showed that the use of GUM significantly reduced the risk of insufficiencies of α-linolenic acid, iron, vitamin C and vitamin D that were associated with the consumption of cow's milk only. A report from Germany has described the similarities and differences between the contribution of 200 ml GUM and 200 ml cow's milk (1.5% fat) to recommended intakes of energy and macro- and micronutrients (3).

In Ireland, nationally representative data on food consumption in young children are available from the National Pre-school Nutrition Survey (NPNS) (4, 5) which was carried out in 2010–11. The aim of the present study was to use data from the NPNS to compare the nutritional adequacy of two groups of 12–24-month-old Irish children by type of milk consumption: GUM together with cow's milk or cow's milk only.

## Experimental methods

### Study groups

Analyses were based on data from the Irish NPNS cross-sectional food consumption survey conducted in 2010–2011 to establish a database of habitual food and drink consumption in a representative sample of children aged 12–59 months (*n*=500). A quota sampling approach was adopted using the most recently published Irish census (6) to achieve a sample of 125 children within each of four age groups (12–23 months, 24–35 months, 36–47 months and 48–59 months) with 50:50 male/female representation in each group. Children were recruited from a database of names and addresses of children compiled by ‘eumom’ (an Irish parenting resource) (www.eumom.ie) or from randomly selected childcare facilities in selected locations. Written informed consent was obtained from the parents/guardians of each child that participated in the survey. The study was carried out according to the guidelines laid down in the Declaration of Helsinki, and all procedures were approved by the Clinical Research Ethics Committee of the Cork Teaching Hospitals (Ref: ECM 4 (a) 06/07/10). Further details of the survey methodology are available at www.iuna.net.

Data for the present study were included from children aged 12–24 months with an average daily total milk intake of at least 300 g and consuming GUM (≥100 g/day) together with cow's milk (consumers of GUM; *n*=29) or cow's milk only (non-consumers of GUM; *n*=56). Children who were breast fed or consuming follow-on formula were excluded.

Consumers of GUM were similar to non-consumers with respect to age (consumers of GUM 16.3 months; non-consumers of GUM 18.2 months) and parental socio-economic status (SES) (professional workers: consumers of GUM 72%, non-consumers of GUM 73%; non-manual workers: consumers of GUM 14%, non-consumers of GUM 14%; manual workers: consumers of GUM 13%, non-consumers 13%). However, the two groups differed from the general population of children under the age of 15 with regard to parental SES (professional workers: 53%; non-manual workers: 25%; manual workers: 22%) (6).

### Food intake assessment

A 4-day weighed food diary was used to collect detailed food and beverage intake data. In all cases, the study period included at least one weekend day. The researcher made three visits to the participant and his/her caregiver during the 4-day period: an initial training visit to show how to keep the food diary and use the weighing scales; a second visit 24–36 h into the recording period to review the diary, check for completeness and clarify details regarding specific food descriptors and quantities; and a visit 1 or 2 days after the recording period to check the final days and to collect the diary. Caregivers were asked to record detailed information regarding the amount, type and brand of all foods, beverages and nutritional supplements consumed by the child over the 4-day period and where applicable the cooking method used, the packaging size and type and details of recipes and any leftovers.

A hierarchical approach to food quantification was used as follows:

1. Weighed (participant/manufacturer weights) – a portable food scales (Tanita kd-400, Japan) was provided and the caregiver was given detailed instructions (including a demonstration) on how to use the food scales. This method was used to quantify 78% of foods and drinks consumed. A further 7% of weights were derived from manufacturer's weights. To facilitate the collection of such data, caregivers were asked to collect all packaging of food and beverages consumed by the child in a storage bag provided.
2. A photographic food atlas for pre-school children (7) was used to quantify 6% of foods and beverages consumed.
3. A database of average portions of certain foods was compiled by the research team and was used to quantify 0.5% of foods and beverages consumed.
4. Food weights and average portions of foods estimated by the Ministry of Agriculture, Fisheries and Food (MAFF) (8) were used to quantify 1% of foods and beverages consumed.
5. Household Measures such as teaspoon, tablespoon, and so on, were used to quantify 6% of foods and beverages consumed.
6. The researcher estimated portion sizes based on the child's previous eating patterns. This method was used to quantify 1.5% of foods and beverages consumed.

### Estimation of nutrient intakes

Nutrient intakes were estimated using WISP^©^ (Tinuviel Software, Anglesey, UK), which uses data from *McCance and Widdowson's the Composition of Foods*, fifth and sixth editions plus all nine supplemental volumes to generate nutrient intake data, as described elsewhere (5). During the NPNS, modifications were made to the Irish Food Composition Database (9) to include all recipes of composite dishes, nutritional supplements, generic Irish foods that were commonly consumed, new foods on the market and all infant/toddler foods and milks that were consumed during the survey period. Information on brands was also recorded.

### Comparison of nutrient intakes with dietary reference values

Mean Daily Intakes (MDI) for carbohydrate and fat were compared to reference intake ranges recommended by EFSA for carbohydrate (45–60% energy (%E) from age 1 year) (10) and for total fat (35–40% energy (%E) in the second and third year of life) (11). For dietary fibre, MDI were compared to the adequate intake of 2 g/MJ as recommended by EFSA (10), while for protein, MDI were compared to the average requirement and the population reference intake (PRI) of 0.95 and 1.14 g/kg body weight per day, respectively, for 1-year-olds and 0.85 and 1.03 g/kg body weight per day, respectively, for 1.5-year-olds derived by EFSA (12).

Estimated Average Requirements (EAR) as established by the Department of Health (UK) (13) were used as cut-offs to estimate the proportion of children with inadequate intakes of micronutrients (calcium; iron; zinc; vitamin A, C, B6, B12, folate, thiamine, riboflavin and niacin). This method has been shown to be effective in obtaining a realistic estimate of the prevalence of dietary inadequacy (14). For vitamin D, MDI were compared with the American Institute of Medicine (IOM) EAR (15) and the UK recommended nutrient intake (RNI) (13).

The risk of excessive intake of micronutrients was evaluated by comparing MDI to the Tolerable Upper Intake Level (UL). The UL is defined as the maximum level of total chronic daily intake of a nutrient (from all sources) judged to be unlikely to pose a risk of adverse health effects in humans (16). Intakes were compared to respective ULs derived by EFSA/EU Scientific Committee for Food for vitamin D (17), retinol (18), vitamin B6 (19), folic acid (20), zinc (21) and by the Food and Nutrition Board in the United States for calcium (15), iron (22), and vitamin C (23).

### Under-reporting

Data were analysed including and excluding under-reporters. Minimum energy intake (EI) cut-off points, calculated as multiples of Basal Metabolic Rate, were used to identify under-reporters of energy (24, 25). Data shown include under-reporters (7%), as their removal did not change the overall trends observed.

### Statistical analysis

Data analysis was conducted using PASW^©^ for Windows version 18.0 (SPSS Inc., Chicago, IL, USA). Independent *t*-tests (parametric data) or the corresponding Mann–Whitney tests (non-parametric data) were used to assess differences between energy and nutrient intakes of consumers and non-consumers of GUM.

## Results

The nutritional composition of GUM available on the Irish market (three brands, for children aged 1 year and over) and the average composition of whole cow's milk are shown in Table 1. The two brands that are predominantly consumed by Irish children had identical composition. Compared to whole cow's milk, the GUM have similar energy and fat content, higher ratio of unsaturated to saturated fat, higher carbohydrate (lactose), and lower protein. Two brands contained dietary fibre (galacto-oligosaccharides/fructo-oligosaccharides). For micronutrients, the most marked differences were for iron and vitamin D for which (unfortified) cow's milk contained very little while GUM contained nutritionally significant amounts.

**Table 1:** Nutritional composition of GUM and whole cow's milk

	Composition per 100g	
	
	Whole cow's milk^1^	GUM^2^
Energy (kJ)	274	274–289
Protein (g)	3.3	1.5–1.8
Fat (g)	3.5	3.0–3.3
of which saturated (g)	2.2	0.8–1.3
of which unsaturated (g)	1.3	2.0–2.2
Carbohydrate (g)	4.5	7.4–8.5
Dietary Fibre (g)	0	0–1.2
Sodium (mg)	43	26–30
Calcium (mg)	118	78–86
Iron (mg)	0.03	1.2
Zinc (mg)	0.4	0.9
Thiamine (mg)	0.03	0.05–0.1
Riboflavin (mg)	0.23	0.11–0.14
Vitamin B6 (mg)	0.06	0.04–0.06
Vitamin B12 (µg)	0.9	0.14–0.18
Total Niacin (mg)	0.8	0.4–0.5
Folate (µg)	8	12–13
Retinol (µg)	30	65–70
Vitamin D (µg)	Trace	1.5–1.7
Vitamin C (mg)	2	12–15

1 McCance & Widdowson Composition of Foods—updated for total fat and saturated fat from Irish composition data.

2 Manufacturer's information, range based on three products from two manufacturers.

The mean daily intake of total milk in consumers of GUM (558 g) was higher than in non-consumers of GUM (480 g); however, the difference was not statistically significant. In consumers of GUM, the mean daily intake of GUM was 386 g and the average contribution of GUM to total milk intake was 60%.

MDI of consumers and non-consumers of GUM were similar for energy, total fat, sodium, calcium, thiamine, riboflavin, niacin, folate, and vitamin A. Compared to non-consumers, consumers of GUM had significantly higher intakes of carbohydrate, dietary fibre, iron, zinc, vitamin C, and vitamin D, and lower intakes of protein, saturated fat, vitamin B6, and vitamin B12 (Table 2).

**Table 2:** Mean daily energy and nutrient intakes in Irish children aged 12–24 months by GUM consumer group

	Consumers of GUM (*n*=29)	Non-consumers of GUM (*n*=56)	*P*
Energy (MJ)	4.4	4.3	0.692
**Protein (g)**	**38.1**	**43.7**	**0.020**
Fat (g)	39.3	40.2	0.740
**Saturated fat (g)**	**16.1**	**20.3**	**0.002**
**Carbohydrate (g)**	**134.6**	**123.2**	**0.025**
**Protein (%TE)**	**14.3**	**17**	**0.000**
Fat (%TE)	33.2	35.1	0.089
**Saturated fat (%TE)**	**13.5**	**17.8**	**0.000**
**Carbohydrate (%TE)**	**48.4**	**44.6**	**0.010**
**Dietary fibre (g)**	**13.2**	**10.2**	**0.000**
Sodium (mg)	840	1008	0.078
Calcium (mg)	902	996	0.086
**Iron (mg)**	**10.4**	**5.9**	**0.000**
**Zinc (mg)**	**7.3**	**5.1**	**0.000**
Thiamine (mg)	1	1	0.742
Riboflavin (mg)	1.7	1.9	0.080
**Vitamin B6 (mg)**	**1.2**	**1.4**	**0.003**
**Vitamin B12 (µg)**	**3.6**	**5.4**	**0.000**
Total niacin (mg)	17.5	18.1	0.442
Folate (µg)	169	174	0.420
Vitamin A (µg)	969	759	0.088
**Vitamin D (µg)**	**9.2**	**2.1**	**0.000**
**Vitamin C (mg)**	**118**	**58**	**0.000**

Bold denotes significantly (*P*<0.05) different nutrient intakes between GUM consumer groups.

**Fig. 1:**
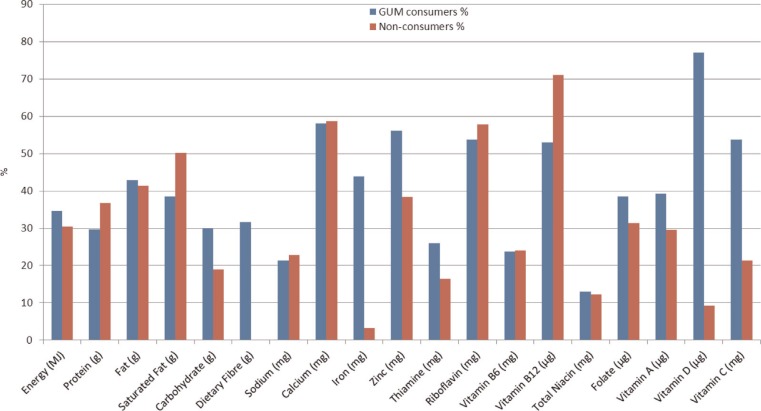
Contribution of total milk to mean daily energy and nutrient intakes in Irish children aged 12–24 months by GUM consumer group.

Milks were a significant source of energy for both consumers and non-consumers of GUM, contributing on average 31–35% of total energy intake (Fig. 1). Milks also made a significant contribution in both groups to intakes of macronutrients and a range of micronutrients, being more marked for consumers of GUM for dietary fibre, iron, vitamin C, and vitamin D.

For both consumers and non-consumers of GUM, mean protein intake was 3.4–3.6 g/kg body weight per day (equivalent to about three times the PRI) and there were no children with intakes of protein lower than the EAR indicating that protein intakes were adequate. Mean fat intakes were 33–35% energy and most children in both groups had fat intakes less than 40%E (93% of consumers of GUM, 80% of non-consumers of GUM). For carbohydrate, mean intakes were 45–48% energy, and consumers of GUM were more likely to have intakes greater than 45%E than non-consumers of GUM (79% vs. 57%, respectively). Mean dietary fibre intakes were 2.4–3.1 g/MJ and most consumers of GUM (93%) and non-consumers of GUM (77%) had intakes of dietary fibre greater than 2 g/MJ. For both groups, there were very few children with intakes below the EAR for any micronutrient (calcium; zinc; vitamin A, C, B6, B12 folate, thiamine, riboflavin, niacin) except iron and vitamin D. For iron, 59% of non-consumers of GUM had intakes below the EAR but there were no children with intakes lower than the EAR among consumers of GUM. A high proportion of children in both groups had intakes of vitamin D below the IOM EAR of 10 µg/day (consumers of GUM: 69%, non-consumers of GUM 98%). Consumers of GUM were less likely than non-consumers of GUM to have vitamin D intakes below the UK RNI of 7 µg/day (31% consumers of GUM; 95% of non-consumers of GUM). A small number of children across the two groups exceeded the UL for zinc, retinol, and folic acid, but this was associated with consumption of GUM only for zinc.

## Discussion

In this study of children aged 12–24 months, GUM were typically consumed in addition to whole cow's milk, contributing an average of 60% of total milk in consumers of GUM. In the NPNS (2010–11), GUM were reported to be consumed by 25% of children aged 12–24 months in Ireland where whole cow's milk was most widely consumed (88% consumers) and other milks consumed were reduced fat cow's milk (14%), breast milk (7%), follow-on formula (6%), and soya/rice milk alternatives (2%) (4). In that study, consumption of GUM was less common in children aged 25–36 months (14% consumers) (26).

The study shows the importance of milks as a food group in the diets of young children. In both consumers and non-consumers of GUM, total milks represented 31–35% of total energy intake and contributed significantly to dietary intakes of macronutrients and a range of micronutrients. While average total daily energy intakes were similar in both groups, intakes of protein, saturated fat, and vitamins B6 and B12 were lower, and intakes of carbohydrate, dietary fibre, iron, zinc, vitamins C and D were higher in consumers of GUM. These differences in nutrient intakes are largely attributable to the differences in composition between GUM and cow's milk.

For both consumers and non-consumers of GUM, intakes of carbohydrate and fat were generally in line with reference intake ranges recommended by EFSA and intakes of protein and dietary fibre were adequate according to EFSA's recommendations. Intakes of micronutrients were generally adequate, except for iron and vitamin D.

For children consuming cow's milk only, a high proportion had intakes of iron that were below the EAR but there was no evidence of inadequate intakes among consumers of GUM. For vitamin D, almost all children consuming cow's milk only failed to achieve the IOM EAR of 10 µg/day or the UK RNI of 7 µg/day. However, for consumers of GUM, the proportions of children not achieving these reference intakes, while still significant, were much lower than in non-consumers of GUM. Iron and vitamin D are recognised as nutrients for which inadequate intakes have been reported in young children, and there is biochemical evidence of insufficiency for both these nutrients in this age group in European countries (27–30). Furthermore, consumption of milk fortified with iron and vitamin D has been shown to improve body stores for these nutrients in healthy 12- to 20-month-old toddlers (31, 32).

A small number of children across the two groups exceeded the UL for zinc, retinol, and folic acid, but this was associated with GUM consumption only for zinc. Because of the way in which UL has been set for these nutrients in children (i.e. estimated on the basis of body weight or body size from adult values derived using large uncertainty factors) (18, 20, 21), there is little risk of adverse effects occurring in the small proportion of individuals exceeding the UL by a modest amount.

There are similarities between the results of the present study and a recent study (2) in French children aged 1–2 years. That study also showed that consumption of GUM significantly reduced the risk of insufficiencies of iron and vitamin D, as well as of α-linolenic acid and vitamin C compared to French nutrient reference values.

A limitation of the present study is that it is based on small study groups of higher SES than the general population. However, the groups were similar to each other in terms of their mean age and socio-economic grouping allowing reliable comparisons to be made regarding the nutritional adequacy of the two groups.

## Conclusions

In our study of Irish children aged 12–24 months, GUM were typically consumed in addition to whole cow's milk, contributing an average of 60% of total milk in consumers of GUM. Like cow's milk, GUM contributed significantly to intakes of energy, macronutrients, and a range of micronutrients. The main nutritional advantages of GUM consumption are in reducing risk of inadequacies of iron and vitamin D, two nutrients frequently lacking in the diets of young children consuming unfortified cow's milk only.
